# Do intuitive and deliberate judgments rely on two distinct neural systems? A case study in face processing

**DOI:** 10.3389/fnhum.2015.00456

**Published:** 2015-08-25

**Authors:** Laura F. Mega, Gerd Gigerenzer, Kirsten G. Volz

**Affiliations:** ^1^Werner Reichardt Centre for Integrative Neuroscience, TuebingenGermany; ^2^Graduate School of Neural and Behavioral Sciences | International Max Planck Research School, TuebingenGermany; ^3^Max Planck Institute for Human Development, BerlinGermany

**Keywords:** intuition, deliberation, unimodel, dual-system model, orbitofrontal cortex

## Abstract

Arguably the most influential models of human decision-making today are based on the assumption that two separable systems – intuition and deliberation – underlie the judgments that people make. Our recent work is among the first to present neural evidence contrary to the predictions of these dual-systems accounts. We measured brain activations using functional magnetic resonance imaging while participants were specifically instructed to either intuitively or deliberately judge the authenticity of emotional facial expressions. Results from three different analyses revealed both common brain networks of activation across decision mode and differential activations as a function of strategy adherence. We take our results to contradict popular dual-systems accounts that propose a clear-cut dichotomy of the processing systems, and to support rather a unified model. According to this, intuitive and deliberate judgment processes rely on the same rules, though only the former are thought to be characterized by non-conscious processing.

## Introduction

The face reveals the state of the heart.

Dante Aligheri

Imagine the genuine smile spreading across a father’s face as he holds his newborn child in his arms for the first time. To intuitively judge this smile as an honest display of bliss seems elementary. Now picture a young bride-to-be, smiling politely as she receives her mother’s outdated wedding gown to wear at her own wedding. Intuitively judging this smile as dishonest does not pose a big challenge (for most). At times people make judgments intuitively, that is, “quickly and effortlessly, seemingly popping out of nowhere, without much conscious awareness of their origins or of the manner of their formation” (Kruglanski and Gigerenzer, 2011, p. 97). At other times, people arrive at their judgments using deliberation, requiring a higher cognitive load and relying on a thorough thought process accessible to (conscious) awareness. Ekman (2006), one of the pioneers of the field of emotional face perception, devised an example of this: he constructed a training tool to teach people how to detect miniscule changes in facial configuration so that they could learn to distinguish an honest from a deceitful expression. Judging facial expressions is part and parcel of our daily interactions, and their correct interpretation is essential for life in social groups. At times, making judgments to glean meaning from the expressions on faces of others seems intuitive (e.g., in everyday face-to-face communication); at other times, it can be deliberate (e.g., when training to recognize deception, as described above). So-called dual-systems accounts propose that two qualitatively and architecturally distinct cognitive processing systems underlie judgments in one form or another (e.g., Kahneman, 2003; Evans, 2008) In contrast, unified approaches propose that intuitive and deliberate judgments rely on the same (or similar) rules, while differing only along a dimension of consciousness (e.g., Kruglanski and Gigerenzer, 2011). Both conceptualizations and the neural predictions that can be derived from them shall be explained in detail below. The present contribution set out to test whether one of the two kinds of approaches better accounts for the neuroimaging data in a face perception task. We chose to use a brain imaging technique to probe these concepts of cognitive judgment strategies, since functional magnetic resonance imaging (fMRI) is a method ideally suited to compare and contrast neural underpinnings of cognitive processes. In other words, we tested whether—and if so, in what way—the neural substrates underlying a fast and subconscious processing strategy (intuitive) differ from those at the basis of a deliberate one, in an expression authenticity judgment task. Therein participants of instruction groups (“intuitive”/“deliberate”) were presented with positive and negative emotional facial expressions of men and women of different age groups. Participants’ task was to judge the authenticity of these emotional facial expressions as “authentic” or “not authentic.”

Over the last three decades, the number of theoretical frameworks suggesting that judgments can be formed via two qualitatively distinct processes or systems has grown significantly (Sloman, 1996; Stanovich and West, 2000; Kahneman, 2003; Strack and Deutsch, 2004; Evans, 2008). Many of these dual-systems accounts have attributed specific, dissimilar qualities to intuitive and deliberate judgments. Intuitive judgments are assumed to be quick, associative, not conscious, effortless, heuristic, and—by some—error prone. Deliberate judgments, on the other hand, are in much of this research assumed to be just the opposite; particularly, they are assumed to be slow, rule-based, conscious, effortful, analytic, and rational. The literature on judgment and decision-making in (cognitive) psychology, as well as recently in neuroscience and neuro-economics, is abundant with empirical findings interpreted in support of the dualistic paradigm (e.g., McClure et al., 2004; Lieberman, 2007); this trend has not gone unchallenged, however. Keren and Schul (2009, p. 546), for example, call for judgment and decision-making researchers to explore “the natural complement of dual-system theories, namely, a unimodel.” Kruglanski and Gigerenzer (2011), accepting that invitation, suggest a unified theory of judgment, an “adaptive toolbox” of heuristics and other rules. According to their proposed framework, both intuitive and deliberative judgments are in fact based on rules and, crucially, the very same rules can underlie both sorts of judgment. One example is the recognition heuristic, which describes how people choose options they recognize over unrecognized ones: in the classic “city size task,” participants may employ the recognition heuristic by judging one of the two presented cities as larger in population size because they recognize its name. Thus the decision criterion (recognition heuristic) can be used intuitively [i.e., without (consciously) deliberating about the correlation between recognition and criterion, in this case, city size]. Yet the same heuristic can also be used deliberatively (i.e., with forethought and cognitive effort), pondering, for example, on that same correlation, but perhaps as a strategy for investing in stocks (Goldstein and Gigerenzer, 2009). Thus the processes are not clearly separate judgment mechanisms; instead, the crucial distinction between the two processes becomes whether a decision process is used consciously, as in deliberate judgments, or non-consciously, as in intuitive judgments. Put differently, in the adaptive toolbox framework, the term “unimodel” does not mean to imply that there is only one process, but that there is a repertoire of several processes, which can be and are used, consciously and unconsciously, in both judgment types (Gigerenzer et al., 2011). Note that the intuitive decision maker is not aware of the ongoing cognitive processes, in the sense that she can report on them – neither on the cue(s) she is using (e.g., recognition) nor on the way in which the cue(s) are processed – she is, however, aware of a feeling indicating which option to choose or which action to pursue. This consciously experienced (subjective/gut) feeling is suggested to result from the ongoing cognitive processes and signifies intuitive judgments (Volz and Zander, 2014).

We believe that this makes a task requiring participants to judge emotional facial expressions a prime example to examine the mental mechanisms underlying intuitive and deliberate judgment strategies and directly test the neural predictions derived from dual-systems theories as well as the unimodel.

If the dual-systems assumption more closely represents a mapping of an individual’s judgment strategy, we would expect to find architecturally distinct neuro-cognitive processing systems for the two sorts of judgments: following the “Social Cognitive Neuroscience” approach (Lieberman et al., 2002; Lieberman, 2007), we would expect to see activation within the amygdala, basal ganglia, and lateral temporal cortex for participants being instructed to perform the task intuitively, since these neural structures have been suggested to sub-serve non-conscious, implicit, or intuitive cognitive processes (called “X-system,” for reflexive, by Lieberman). In contrast, for participants being instructed to perform the task deliberately, we would expect to see activation within the anterior cingulate cortex, the prefrontal cortex, and the medial temporal lobe (including the hippocampus), since these structures have been suggested to sub-serve conscious, explicit, or rational cognitive processes (which comprise the so called “C-system,” for reflective; Lieberman, 2007). If, however, a unified model such as the adaptive-toolbox framework more closely represents a mapping of people’s reasoning processes, we would instead expect to find overlapping neural networks, with the intuitive judgment group additionally activating areas that have been suggested to reflect unconscious cognitive processing. Given previous findings on intuitive judgments, especially in the perceptual domain (Bar et al., 2006; Volz and von Cramon, 2006; Volz et al., 2008; Horr et al., 2014), we hypothesize these additionally activated areas to be within the medial orbitofrontal cortex (OFC), extrastriate visual areas (BA 19), and infero-temporal cortices. According to a preliminary neuro-cognitive model of intuition, it is suggested that the OFC serves as a rapid detector and predictor of potential content (authenticity, in our case), sending this initial signal via extrastriate areas to inferior temporal areas to enable quick, informed judgments. Furthermore, activation within the anterior insula may specifically be observed for intuitive judgments, given that this area has been suggested as a unique neural substrate instantiating all subjective feelings (Critchley et al., 2004; Craig, 2009), thus/therefore possible also (gut) feelings signaling the outcomes of non-conscious (knowledge-based) cognitive processes.

Given that neuroimaging is ideally suited to assessing similarities and differences on a cognitive level, we utilized this method as a way to directly test the two opposing conceptualizations of intuition and deliberation, namely a dualistic versus a unified approach. To come one step closer to a natural setting in which people in their every-day lives in fact rely on intuitive and/or deliberative judgments, we chose to veer off the often chosen path^1^ of artificial tasks traditionally used to probe decision-making. The task we asked participants to perform is one people perform 1000-fold on a daily basis: judging the facial expression of people. In this manner, we hope to come one step closer to gaining an insight into the neural underpinnings of intuitive and deliberate judgment strategies employed in a naturalistic setting. (Although we do concede that interpreting facial expressions “in the wild” would surely be even more ideal, this is unfortunately rather inconceivable for a neuro-scientific investigation.)

We conducted a fMRI study with a between-subject design, using a task in which participants were shown a series of happy and fearful facial expressions (of young, middle-aged, and older males and females); we then asked the participants if they perceived the expression to be authentic or not. We either instructed participants to base their judgments on their first impression and gut feeling (group intuitive) or on a deliberate strategy of taking into account the position of the facial muscles in the eye and mouth region (group deliberate; see below for detailed instructions). Focusing on the eye and mouth region causes participants to attend to the facial features attended to most (as shown by several eye-tracking studies, e.g., Saether et al., 2009). Thus, the fixation strategy the deliberate group was instructed to use was not arbitrary but resembled the natural scan path of human participants. The direct instruction of decision mode in a between-subject design follows the methodological recommendations of leading experts in the field (Horstmann et al., 2009): by using a between-subject design with the same decision task and dependent variables for both the intuitive and the deliberate condition, the effect of decision mode can truly be investigated without carry-over effects. Although decision time is a characteristic marker of decision mode according to much of the literature, we refrained from using direct time constraints to induce automaticity, since this can lead to divided attention in participants (resulting in introspective questions, such as “How fast am I this time” or “How much time is left”). Instead, as is frequently the case in the literature of judgment and decision-making and also recommended by Horstmann et al. (2009), decision time was used as a marker to check the instruction manipulation.

(Statistical) Tests designed to compare the theoretical underpinnings (i.e., unified model or dual-systems account) of the intuitive and deliberate judgment processes cannot simply be based on contrasting the two processes. Such a contrast can only reveal significant differences between the two kinds of judgment but cannot reveal overlapping activation patterns or gradients of activation. Thus the crucial analysis to test for a unified model or a dual-systems account is a conjunction between the two judgment groups. A conjunction logic is defined as an “and” between truth statements (Nichols et al., 2005). In neuroimaging, this type of analysis reveals brain areas activated in both groups for the contrasts compared. Since the dual-systems approach assumes a functional distinction between the two judgment processes, evidence for the veracity of the dual-systems account would paradoxically lie in an “empty” conjunction [i.e., no (task-relevant) brain areas activated by either group]. Conversely, finding commonly activated brain areas for both the intuitive and the deliberate groups would speak against such a model. Furthermore, since the reaction time (RT) of the participants can be taken as an objective marker of their adherence to the instructed judgment strategy, as shown previously (Bolte and Goschke, 2005; Ahlgrimm and Glöckner, 2009), an analysis incorporating each participant’s mean RT as indicator of strategy compliance can be used as a second approach for comparing qualitative or quantitative differences in judgments. If different brain areas were activated in each of the two processes, with the number of areas and the intensity of their activation varying as a function of speed, we would consider this as evidence supporting the dual-systems account. That is, fast responding individuals in the intuitive group would show more activation foci or more enhanced activation within the X-system, whereas fast responding individuals in the deliberate group would show fewer or less activated foci within the C-system (since the dual-systems account conceptualizes deliberation as a slower process than intuition).

If the two processes are not inherently distinct but rather deviate only on certain dimensions (such as consciousness) as a unified model would suggest, we might well see the same or similar kinds of brain areas activated for both processes in a covariate analysis; with the expansion (i.e., the number of areas) or the intensity of activation varying as a function of RT (i.e., strategy adherence). Put differently, fast responding individuals in both of the groups would show similar activation patterns to those of slow responding individuals in both groups. Additionally, in keeping with the consciousness dimension as a differentiating category, we would expect an incorporation of brain regions known to be important for non-conscious processing (i.e., medial OFC and anterior insula), for the intuitive judgment process.

To reiterate, we set out with this work to directly test the currently held dualistic model of judgment and decision-making in an everyday type of task, namely the judgment of the authenticity of facial expressions, via fMRI. We compared the judgment processes of intuition and deliberation with two differently instructed participant groups, subsequently analyzed their neural responses in conjunction and covariate analyses, and also contrasted them directly with each other. We thereby address the current debate on whether these two processes represent two separate systems, relying on distinctly different neural pathways; or whether they are rather two sides of a coin, diverging along a dimension of consciousness.

## Materials and Methods

### Participants and Instruction

Thirty healthy, right-handed volunteers were included in this study (14 females; mean age = 26.75, SD = 3.2, range = 22–39). Two participants (one male, one female) were excluded from analysis because of technical difficulties during scanning. We obtained informed consent from each participant prior to the experiment according to the Declaration of Helsinki. The local ethics committee of the “Charité Universitätsmedizin” Berlin approved the experimental standards. Data was handled anonymously. All participants were native German speakers, had no history of neuropsychiatric disorders, and were not currently taking psychoactive medications. The behavioral performance was measured via RT.

Participants were pseudo-randomly assigned to the two instruction conditions: in the intuitive instruction group 14 participants (seven females, mean age 26.4) received the following instruction:

“Your task is to judge the emotional expression you will see with regard to its authenticity (realness)…Previous studies have shown that people are good at judging the authenticity (realness) of a smiling or fearful expression if they follow their initial feeling, that is, answer spontaneously and without thinking for too long. We therefore ask you to make your judgment quickly and most importantly, to follow your first feeling, thus deciding ‘based on your gut.”’

The term “intuition” was intentionally not used in the instruction in order to avoid bias effects. In contrast, the instruction for the 14 participants (six female, mean age 26) in the deliberate instruction group was as follows:

“Your task is to judge the emotional expression you will see regarding its authenticity (realness)…Previous studies have shown that people are good at judging the authenticity (realness) of a smiling or fearful expression if they analyze and study the expression well, that is, think about their answer. Therefore, before you respond, study the expression thoroughly—within the given time. Most importantly, pay attention to the matching of the facial muscles in the eye and mouth regions.”

A major emphasis of this work was to investigate (intuitive and deliberate judgments) in an every-day type task setting, as stated previously. Therefore, although rating the authenticity of facial expressions may at first glance seem ambiguous, this actually very well-reflects the real-life situation: in a natural setting, we very rarely get feedback about the communicative content of another person’s expression. Depending on the experiences we’ve had in our lives (i.e., our social surroundings and upbringing, as well as our cultural background), we may tend to be very suspicious and fear that others are deceiving us, or we may tend to be very trusting and take everything—literally—at “face value.” Our main research question pertains not to the correctness or a person’s answer but rather differences in the neural activations of the two judgment strategies. We therefore decided considered the importance of a task that was as naturalistic as possible (in an fMRI study design) high enough to tolerate the trade-off of using a somewhat ambiguous question.

### Task Outline

**FIGURE 1:**
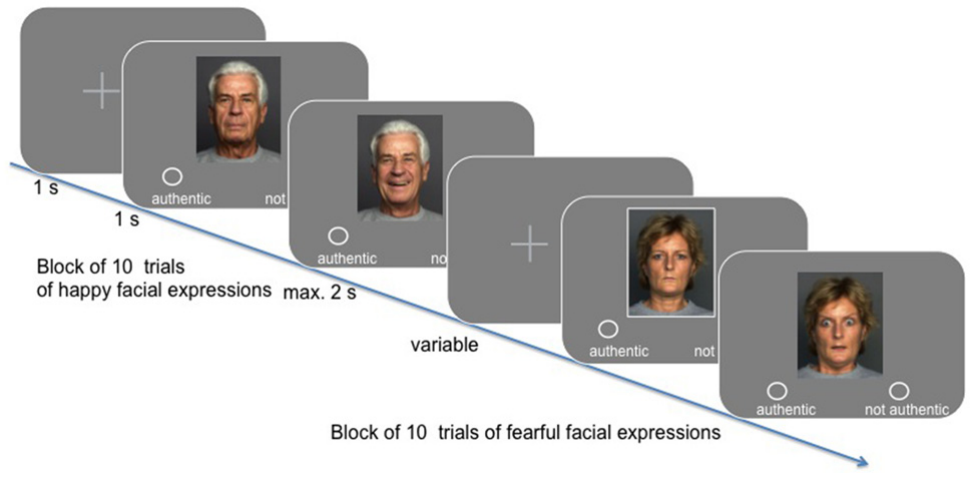
**Timeline overview of the task design using exemplary stimuli from the FACES database kindly provided by Ebner et al. (2010)**.

In the experimental session, participants were presented with 340 stimuli, showing either a happy or a fearful facial expression, while their hemodynamic activity was measured (see **Figure 1**). Their task was to indicate whether they perceived the facial expression to be authentic or not (yes/no response assignment was balanced across participants). Stimuli were taken from the FACE database established by Ebner et al. (2010). Specifically, we presented 170 happy and 170 fearful facial expressions, wherein gender and age group [“young” (*M* = 24.2 years, SD = 3.4; range 19–31), “middle-aged” (*M* = 49.0 years, SD = 3.9; range 39–55), and “57 years and older” (*M* = 73.2 years, SD = 2.8; range 69–80) as classified by Ebner et al., 2010] were balanced across conditions. Happy and fearful facial expressions were presented in blocks of 10, resulting in 34 blocks across the entire experiment (block transition = 6 s). All trials lasted for 6 s (i.e., three scans at TR = 2 s): after a short fixation (variable duration), the neutral facial expression of the respective lay actor was shown for 1 s, followed by the presentation of the emotional facial expression, which was either shown for a maximum of 2 s, or for as long as participants took to make their choice (response-dependent abortion). A fixation cross was presented for the remaining time of the trial.

We randomly varied the onset of each stimulus presentation relative to the beginning of the first of the three scans in order to enhance the temporal resolution of the signal captured (Birn et al., 2002). After the experimental session, participants were asked to fill in a questionnaire asking how difficult they perceived the task to be in general, and whether participants found it difficult to follow the instructed strategy, among other things (see **Table 1** for full questionnaire). Finally, participants were debriefed and thanked.

**Table 1:** Results of the post-session questionnaire.

Question	Intuitive group [%]	Deliberate group [%]
**How difficult was the evaluation of the authenticity of the faces?**		
Very difficult	71.43	64.29
Moderately difficult	7.14	7.14
Easy	21.43	28.57
Very easy	0	0
**Was a specific emotion more difficult?**		
General	100	100
Happy	100	76.9
Fear	0	23.1
**Did one of the presented age groups influence your evaluation? If so, how?**		
Yes	50	85.71
Easier for old	57.14	41.67
More difficult for old	42.86	33.33
Older more often authentic	0	25
**Was it difficult to follow the instructed strategy?**		
Very difficult	15.38	57.14
Moderately difficult	7.69	14.29
Easy	76.92	28.57

*Answers are presented in terms of % of participants per group.*

### MRI Scanning Procedure

Imaging was performed on a 3T scanner (Siemens TrioTim, Erlangen, Germany) equipped with a standard birdcage head coil. 17 axial slices [4 mm thickness, 25% spacing, field of view (FOV) 21 cm, data matrix of 64 × 64 voxels, and in-plane resolution of 3.3 mm × 3.3 mm] covering the whole brain were acquired using a single-shot spin-echo echo-planar imaging (SE-EPI) sequence [TR 2 s, echo time (TE) 80 ms, flip angle 90°] sensitive to blood oxygen level-dependent (BOLD) contrast. Two functional runs with 170 time points each were run with each time point sampling over the 17 slices. Macroscopic field gradients occurring at air-tissue boundaries can cause signal losses in gradient echo sequencing (Miyapuram et al., 2007). Therefore, since activity in the OFC was expected and this area lies near an air-tissue boundary, images were acquired using a spin echo sequence, achieving a higher signal to noise ratio (SNR). Furthermore, geometric distortions due to static magnetic field in-homogeneities were characterized by a field-map scan at the end of each session. Additionally, T1-weighted structural MR images were acquired for the registration of fMRI data to each participant’s standard anatomical space (TR = 1550 ms, TE = 2.34 ms, FoV = 244 mm × 244 mm, voxel size = 1 mm × 1 mm × 1 mm, interslice gap = 0.5 mm).

### Image Processing and Analysis

Functional imaging data was processed and analyzed using FEAT (FMRI Expert Analysis Tool) version 5.98, part of FSL 4.1.9 (FMRIB’s Software Library, http://www.fmrib.ox.ac.uk/fsl), and motion-corrected using rigid-body registration to the central volume (Jenkinson et al., 2002; Woolrich et al., 2004). The brain extraction tool (BET) was used for extraction from skull and surrounding tissue (Smith, 2002). To correct for the temporal offset between the slices acquired in one scan, a (Hanning-windowed) sinc interpolation was applied. To remove low frequency signals, a temporal high-pass filter with a cut-off frequency of 1/100 Hz was used. Spatial smoothing was applied by using a Gaussian filter with 5 mm full-width half maximum (FWHM). Registration of the SE-EPI images to the individual high-resolution structural images and normalization into standard (MNI) space was performed using affine registration as implemented in FLIRT (Jenkinson et al., 2002). To improve the signal quality, especially within areas affected by susceptibility artifacts, a distortion correction procedure was applied using FUGUE. In the first-level analysis, by using FILM (FMRIB’s improved linear model), a general linear model was fitted into pre-whitened data space to account for local autocorrelations in the fMRI residuals (Woolrich et al., 2001). The hemodynamic response function was modeled as a gamma function, with a mean lag of 6 s and SD of 3 s. The events of interest were each modeled with their temporal derivatives. For the decision phase, we modeled the presentation of the emotion-related facial expression as independent events. On the single-subject level, all stimulus-part categories (i.e., “happy,” “fearful,” “female,” “male,” “young,” “middle-aged,” and “older”) were contrasted against the implicit baseline. Z (gaussianized T/F) statistic images were thresholded using clusters determined by *Z* = 2.33 and a (corrected) cluster significance threshold of *P* = 0.05. Thus the trials were classified based on objective stimulus criteria. Subsequently, the resulting contrast-images were introduced into a fixed-effects model to statistically concatenate the two different runs (*Z* > 3.09, *P* = 0.05). Next, two additional higher-level analyses were conducted. The first was used to define contrasts for the GLM of the group analysis. Finally, the third higher-level analysis was performed as a group analysis. Contrasts were defined testing activations found in the intuitive group against those of the deliberate (intuitive versus deliberate) and vice versa (deliberate versus intuitive), as well as both group activations separately against baseline (*Z* > 3.09, *P* = 0.05). Note that we used a Spin-Echo (SE)-EPI sequence, which is sufficiently sensitive for cognitive studies. Its drawback, however, is a lower statistical power of the SE-sequences as compared to Gradient-Echo EPI, corresponding to a reduction in *Z* scores of about a factor of three.

## Results

### Behavioral Results

**FIGURE 2:**
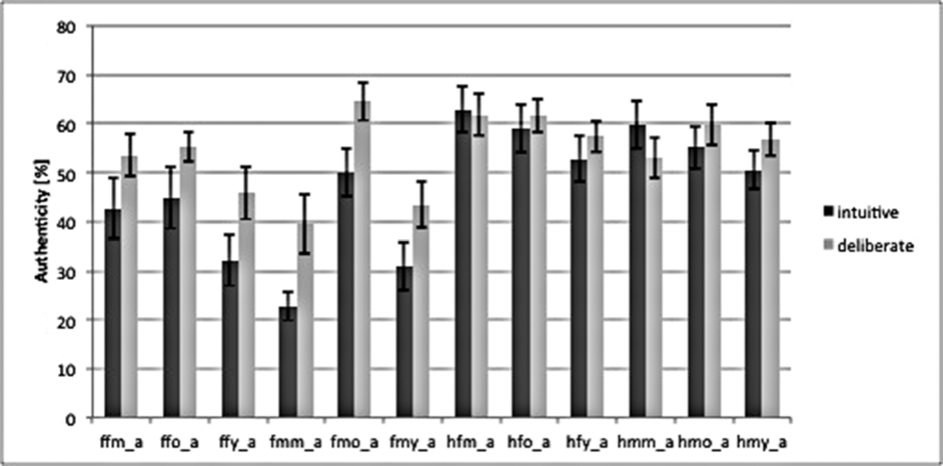
**Bar graph of authenticity judgments for both instruction-groups, based on the combination of categories in the stimulus material**. *ffm_a = fear, female, middle, authentic; ffo = fear, female, old; ffy = fear, female, young; fmm = fear, male, middle; fm0 = fear, male, old; fmy = fear, male, young; Labels for “happy” = h are analogous to “fear.”* Behavioral data was analyzed using repeated measures analysis of variance (rmANOVA) in SPSS (SPSS Statistic 20.0, IBM, Chicago, IL, USA), as well as explorative data analysis. The frequency of behavioral scores (i.e., RT) per cell was checked for Gaussian distribution to confirm the use of rmANOVA for the analysis. Missing values were omitted. Error bars mark SEM.

Our study was conducted with a between-subject design, dividing participants into group intuitive (*n* = 14) and group deliberate (*n* = 14) based on the instructions they were given (see above). A manipulation check of strategy induction using decision times as dependent variable – which has been suggested as a qualified method (De Vries et al., 2008; Horstmann et al., 2009) – revealed that our instructions successfully induced intuitive and deliberate decisions. A repeated measure analysis of variance (rmANOVA) of RTs revealed a main effect of decision mode [*F*_group_ (1,26) = 6.94, *P* = 0.01]. Deciding was faster in the intuitive [1379 ms, 95% confidence interval (CI) = 1105–1652; SE = 133.1] than in the deliberate decision mode (1875 ms, CI = 1601–2148; SE = 133.1). As for the authenticity judgments themselves, there was no difference in the judgments between the intuitive and the deliberate group, i.e., the deliberate group did not rate emotional facial expressions as being more authentic than the intuitive group. This is in line with behavioral predictions derived from the unified model theory. That is, the rates of authenticity judgments did not differ for single stimuli, or for aggregated groups of stimuli or on average (see **Figure 2**).

A post-session questionnaire, in which participants were asked about their difficulties adhering to the instructed strategy, revealed relevant behavioral differences. That is, only one participant from the intuitive instruction group (15.4%) reported overall difficulties with the instruction, but eight participants from the deliberate group (57.1%) did. This difficulty was reflected in their response latencies, as this subset of participants showed the longest RTs (see **Table 1**).

### Neuroimaging Data

We conducted whole-brain analyses rather than restricting our view by focusing on specific regions of interest, since we were interested in all brain areas activated by the judgment task in both of the instructed groups,. On the single-subject level, all stimulus-part categories (dependent variables) were contrasted against the implicit baseline. Subsequent higher-level analyses were conducted in two steps, wherein the individual analysis data were collapsed into group-level contrasts. Thereby, the group-contrasts consisted of the data of 14 participants per group, split into 12 variables (happy, fear, female, male, young, middle, old) with a cluster *Z* threshold of 3.09 and *P* = 0.05 using a fixed-effects model (see Materials and Methods).

#### Analyses Focusing on Reaction Time (Covariate)

Speed has been shown to be one central characteristic of intuitive judgments (Bolte and Goschke, 2005; De Vries et al., 2008; Horstmann et al., 2009). We therefore took participants’ RTs as indicators of strategy adherence (within their respective group). Particularly, the faster participants responded, the more intuitive (in the intuitive group) and the less deliberate (in the deliberate group) their judgment strategy was assumed to be. Since the focus of our study was on judgment processes rather than outputs (the judgments themselves), utilizing a central process property such as speed for our analysis was crucial. Accordingly, modeling the RT as covariate in the group-level fMRI analysis helps to explain variability in the BOLD signal that is uniquely caused by cross-subject differences in strategy adherence and cannot be accounted for by the other regressors. To reiterate, each participant’s mean RT was added to the GLM in this analysis. Contrasts were defined for the group mean activation against baseline, as well as the positive covariant [1] and the negative covariant [-1]. *Z* (Gaussianised T/F) statistic images were thresholded using clusters determined by *Z* > 3.09 and a (corrected) cluster significance threshold of *P* = 0.05 (Worsley, 2001).

##### Mean overall activation

**FIGURE 3:**
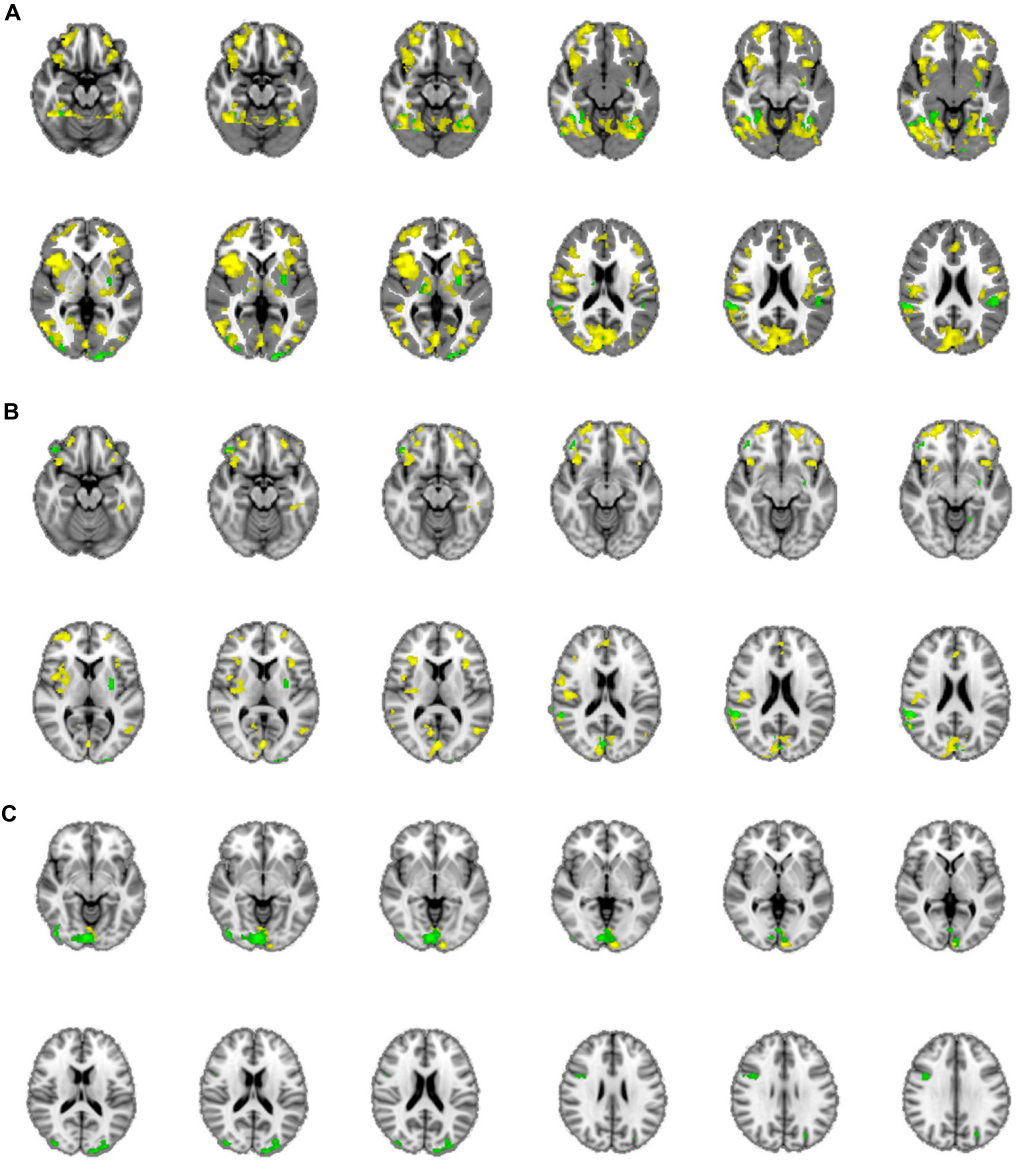
**(A)** Mean group activations: intuitive (yellow): visual areas (lingual gyrus, extrastriate cortex), bilateral OFC, left insula and thalamus, bilateral STG/TPJ, right paracingulate gyrus, and left precuneus. Deliberate (green): visual areas (middle and inferior occipital gyrus, occipital fusiform cortex, fusiform gyrus, and cuneus), STG/TPJ, putamen, thalamus and angular gyrus. **(B)** Activations revealed by the negative covariate (“fast” RT); Intuitive (yellow): TPJ, paracingulate gyrus, precuneus, thalamus, OFC, intraparietal lobula (IPL). Deliberate (green): right OFC and temporoparietal regions (TPJ, angular gyrus, surpamarginal gyrus), lingual gyrus, cuneus. **(C)** Activations revealed by the positive covariate (“slow” RTs); Intuitive (yellow): lingual gyrus/extrastriate cortex. Deliberate (green): visual areas (lingual gyrus, cuneus, inferior lateral occipital gyrus), middle frontal gyrus.

**FIGURE 4:**
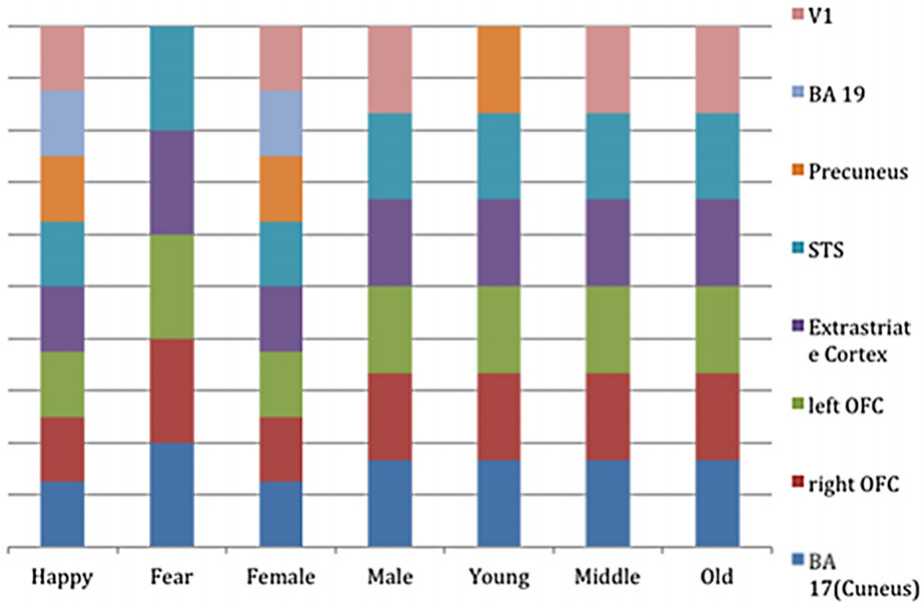
**Bar-graph representing the clusters of activation for the different regressors (i.e., stimulus-part categories).** Height of the bars only indicates activity in this region and is not an indication of amount.

The mean activations of the contrast happy versus basline for the intuitive group is found within visual areas—lingual gyrus, extrastriate cortex—as well as in the bilateral OFC, left insula and thalamus, bilateral superior temporal gyrus (STG)/temporoparietal junction (TPJ), right paracingulate gyrus, and left precuneus (see **Figure 3A**, activations depicted in yellow). For the deliberate group, this analysis revealed mean group activity in visual areas—middle and inferior occipital gyrus, occipital fusiform cortex, fusiform gyrus and cuneus—as well as in the right STG/TPJ, left putamen, right thalamus, and left angular gyrus (see **Figure 3A**, activations depicted in green; see **Tables 2** and **3** for complete list of activations). All other baseline contrasts revealed analogous patterns of activations (see **Figure 4**). For this reason, and because our hypotheses were focused on the between-subject effect rather than the relative contribution of specific stimulus-encoded categories (e.g., emotion, gender), we chose to focus on the “happy” contrast as representative dependent variable for the subsequent analyses.

**Table 2:** Neural activations of the intuitive group as revealed by covariate analysis.

Group mean	Hemisphere	# Voxels	*Z*-MAX	*X*	*Y*	*Z*
Lingual gyrus (BA 18)	bi	11895	8.13	0	-82	8
Insula (BA 13)	L	1945	7.18	-34	18	8
Middle frontal gyrus (and OFC; BA 11)	L	1114	6.58	-28	44	-14
Superior temporal gyrus	L	77	5.59	-52	-26	-2
Middle temporal gyrus	L	118	5.3	-60	-26	26
Middle occipital gyrus (extrastriate; BA 18)	L	111	5.12	-34	-90	6
Medial frontal gyrus	R	381	5.09	4	34	28
Thalamus	R	227	5.05	12	-16	8
Superior occipital gyrus (BA 19)	L	114	4.95	-34	-84	28
Precuneus	L	88	4.82	-14	-58	36
Superior temporal gyurs	R	125	4.28	52	-26	-4
Paracingulate gyrus*	R	79	4.1	2	50	16
**Positive covariate (“slow”)**						
Lingual gyrus	L	227	6.58	-10	-98	-2
**Negative covariate (“fast”)**						
Middle frontal gyrus	L	464	5.68	-26	44	-14
extending into OFC						
Supracalcerine cortex*	R	564	5.44	2	-84	10
Precentral gyrus (BA 44)	R	1286	5.2	48	18	2
Superior frontal gyrus	R	495	5.17	22	62	-10
extending into OFC						
Middle temporal gyrus	L	138	5.06	-48	-64	12
Fusiform gyrus (BA 36)	L	80	4.95	-42	-36	-22
Posterior cingulate (BA 30)	R	99	4.94	10	-68	12
Supramarginal gyrus	R	303	4.88	56	-58	34
Middle frontal gyrus (BA 46)	L	225	4.7	-42	46	0
Insula (BA 13)	L	73	4.65	-34	18	10
Occipital lobe	L	76	4.6	-22	-72	0
OFC (BA 47)	L	80	4.53	-40	18	-10
Medial frontal gyrus	R	71	4.22	4	50	16

Co-ordinates of activations (in mm) are in MNI Space, labels according to Talairach Daemon Labels, unless indicated by an asterisk. *Labels according to the Harvard-Oxford Structural Atlas. L, left hemisphere; R, right hemisphere; bi, bilateral; BA, Brodmann area.

**Table 3:** Neural activations of the deliberate group as revealed by covariate analysis.

Group mean	Hemisphere	# Voxels	*Z*-MAX	*X*	*Y*	*Z*
Occipital lobe	L	285	7.83	-14	-106	-4
Middle occipital gyrus	R	303	6.88	54	-70	-10
Fusiform gyrus (BA 19)	L	381	6.14	-42	-70	-14
Superior temporal gyrus (TPJ, BA 13)	R	328	6.14	58	-42	22
Inferior occipital gyrus (BA 19)	R	160	5.25	44	-78	-2
Insula (BA 13)	L	232	4.93	-52	-30	22
Temporal occipital fusiform cortex*	R	208	4.92	30	-56	-12
Cuneus	R	204	4.84	6	-78	16
Putamen	L	193	4.81	-26	-8	6
Angular gyrus	L	156	4.5	-42	-52	30
Thalamus	R	139	4.36	14	-12	8
**Positive covariate (“slow”)**						
Lingual gyrus (BA 18)	R	471	8.82	6	-88	-6
Inferior occipital gyrus	R	75	7.75	48	-82	-8
Middle frontal gyrus (BA 6)	R	228	6.34	46	6	40
Cuneus	L	350	5.83	-18	-98	18
Lateral occipital cortex	R	90	5.09	36	-88	18
**Negative covariate (“fast”)**						
Occipital lobe	L	81	7.73	-14	-106	-4
Superior temporal gyrus (TPJ; BA 13)	R	136	5.81	58	-42	22
Angular gyrus*	L	180	4.65	-42	-52	30
Supramarginal gyrus (BA 40)	R	92	4.56	62	-54	24
Putamen	L	114	4.51	-28	-8	6
Middle frontal gyrus	R	101	4.47	46	38	-16
extending into OFC(BA 47)						
Cuneus (BA 18)	R	141	4.42	4	-80	18
Lingual gyrus*	L	74	4.14	-18	-58	-4

Co-ordinates of activations (in mm) are in MNI Space, labels according to Talairach Daemon Labels, unless indicated by an asterisk. *Labels according to the Harvard-Oxford Structural Atlas. L, left hemisphere; R, right hemisphere; bi, bilateral; BA, Brodmann area.

##### Negative correlation with RT

A unidirectional analysis investigating brain areas that co-varied negatively with RTs for the intuitive group, revealed extensive areas of activation encompassing right TPJ, paracingulate gyrus, precuneus, thalamus, and right OFC, as well as intraparietal lobula (IPL) and insula, along with areas of visual perception (supracalcarine cortex, fusiform gyrus). It is interesting to note that the analogous analysis (negative co-variation) in the deliberate group exhibited activations in some of the same areas as the intuitive, namely within right OFC and TPJ, as well as within primary and extrastriate visual cortices, but also exhibited activations within dorsolateral prefrontal cortex (dlPFC) and angular gyrus (see **Figure 3B**).

##### Positive correlation with RT

The inverse analysis (positive co-variation) in the intuitive group merely showed activity in the lingual gyrus/extrastriate cortex. We take the activity pattern for the positive co-variation of brain activity within the intuitive participants to show that the people who seemed to adhere less to the instruction — judging from their longer RTs and their answers to the post-session questionnaire — relied mostly on brain regions for visual analysis and did not include the orbitofrontal or temporal structures previously observed to be elicited for this group (see also **Tables 2** and **3** for areas of activation). The test of brain regions showing positive co-variation with RTs in the deliberate group also revealed mostly primary and extrastriate visual areas (inferior and lateral occipital gyrus, cuneus, and lingual gyrus), similar to but more extensive than the activation patterns for the analogous analysis of the intuitive participants (see **Figure 3C**, green activations).

#### Conjunction Analysis

By visual inspection, the two groups show different as well as overlapping activations, the latter being located within posterior-inferior areas. To be reasonably confident that these brain regions are activated across decision mode, we calculated a conjunction analysis of the neural activity revealed for the mean group activations in the covariate analysis, using the baseline contrast “happy” for both instruction groups (cp. Nichols et al., 2005).

**FIGURE 5:**
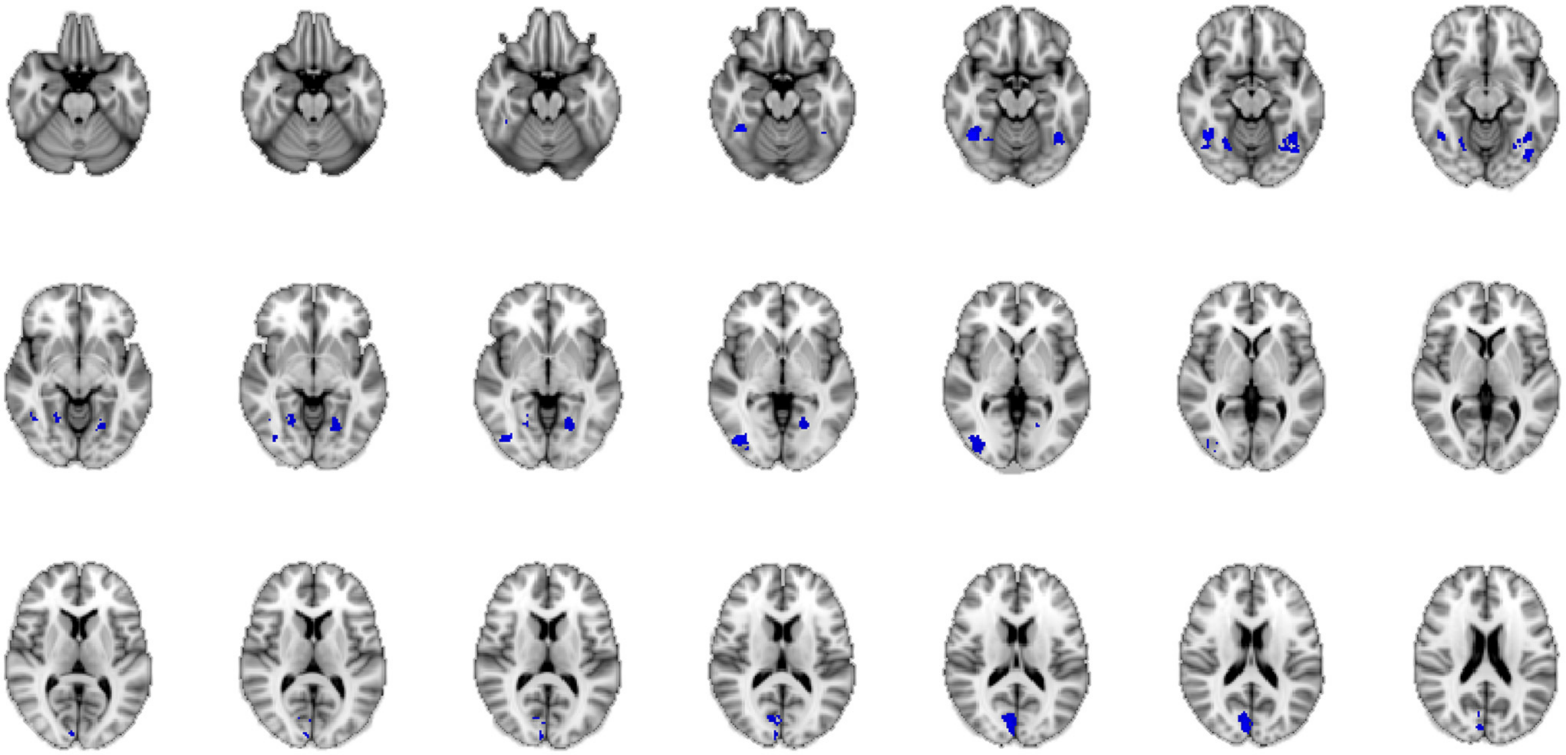
**Neural correlates of the authenticity judgment of happy facial expressions, for both the intuitive and the deliberate instruction group, as revealed by conjunction analysis.** The “core network” of authenticity judgments in this task, as compared to baseline activation is shown through a conjunction of the mean activations in both groups. The blue color of activations was chosen arbitrarily and is not an indicator of de-activation.

A conjunction is defined in logic: “If we have two truth statements A and B, then the conjunction of A and B is true if and only if both A AND B are true” (Nichols et al., 2005, p. 653). In neuroimaging terms this means that the conjunction map shows regions that are commonly activated in the comparison of A and B. In our study, A and B represent the intuitive and the deliberate decision mode. By calculating a conjunction analysis between the group mean results of the baseline contrast of the intuitive group and those of the deliberate group, we were able to uncover the areas activated by the task, regardless of instruction, i.e., the “common network” for the judgment of the authenticity of emotional facial expressions across decision mode. This analysis revealed several clusters of bilateral activity in the fusiform gyrus, the cuneus, and the inferior occipital gyrus. These all belong to the classic core system of face perception (e.g., Fusar-Poli et al., 2009; Atkinson and Adolphs, 2011; see **Figure 5**). Thus for both the intuitive and the deliberate group, a commonly activated brain network was revealed when participants judged the authenticity of emotional facial expressions.

In summation, both the conjunction and the covariate analyses revealed (partially) similar regions of activation for both instruction groups, not two distinctly different brain networks (such as the X- and the C-Systems), as would be predicted by dual-systems theories.

#### Direct Contrast Intuitive versus Deliberate

**FIGURE 6:**
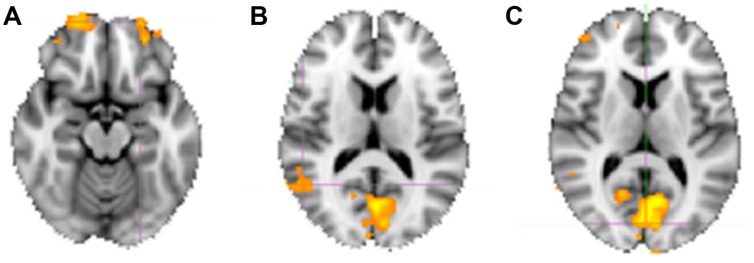
**Neural substrates activated when judging happy faces, contrasting activity of the intuitive versus the deliberate group. (A)** Shows an axial slice view of bilateral activity in anterior portions of the OFC, **(B)** shows the activation in the right STG/TPJ (coordinates), and **(C)** shows the bilaterally activated cuneus (see **Table 4** for co-ordinates).

For the sake of completeness, we additionally directly contrasted the activation pattern of the intuitive group with that of the deliberate group. According to a dual-systems model, one would expect to find distinct activations in a direct contrast: activation within the X-system for intuitive as compared to deliberate decisions, and activation within the C-system for the inverse contrast. A unified account, on the other hand, would only predict differential activation in areas known to sub-serve characteristics of intuition such as non-conscious processing specifically for the intuitive group. The analysis was conducted in multiple levels. First, single-subject analysis GLMs were modeled with seven baseline regressors (“happy,” “fearful,” “female,” “male,” “young,” “middle-aged,” “older”) encoding the stimulus-part categories (i.e., dependent variables), along with their temporal derivatives. On the next level, the regressors of the single-subject analyses were defined as contrasts for the group analysis, allowing subject-level covariations to be entered into the analysis. The final analysis defined contrasts for the comparison of the instruction groups (contrasting the activity of the intuitive group with that of the deliberate one). In a direct contrast of the intuitive group with the deliberate group in the “happy” condition, functional activity was revealed in bilateral cuneus (BA 17), precuneus, bilateral OFC, right fusiform gyrus (BA 18), and right STG (BA 22; see **Figure 6** and **Table 4** for areas of activation). A very similar pattern of activation as revealed by taking into account those activations elicited when viewing and judging stimuli depicting a happy expression (“happy” condition) was also seen for all other dependent variables (see **Figure 4**).

**Table 4:** Neural activations as revealed by directly contrasting activations of both groups.

	Hemisphere	# Voxels	*Z*-MAX	*X*	*Y*	*Z*
**Happy**						
Cuneus	L	86	9.39	-6	-104	8
Lingual gyrus (occipital lobe)	bi	1720	7.38	0	-80	8
Medial frontal gyrus	R	736	6.13	20	62	-2
Superior frontal gyrus (BA 10)	L	271	5.36	-24	62	-16
extending into OFC					
Lingual gyrus (BA 18)	R	157	5.26	26	-74	-8
[fusiform gyrus*]					
Cuneus	R	88	5.12	8	-84	34
Superior temporal gyrus	R	133	4.55	64	-54	14
(BA 22)					
Precuneus (BA 7)	R	122	4.45	8	-62	42

MNI Space co-ordinates of activations (in mm) in the contrast intuitive > deliberate for the specified regressor. Labels according to Talairach Daemon Labels, unless indicated by an asterisk. Clusters shown in descending order of *Z-Max* values. L, left hemisphere; R, right hemisphere; bi, bilateral. *Labels according to the Harvard-Oxford Structural Atlas.

The inverse contrast (deliberate versus intuitive group) did not show any significant differences. This may indicate that the deliberate judgment strategy is actually a sub-version of the intuitive one, lacking activations needed for non-conscious processing. It could also indicate that participants in the deliberate group showed large inter-individual variability in processing. The latter assumption may be supported by our post-session questionnaire data: the deliberate group seems to be composed of (at least) two subgroups, one of which reported difficulties with the instructed deliberate strategy (57%), and the other no difficulties (43%).

## Discussion

Judging facial expressions is essential for natural social interactions. These judgments may be intuitive (e.g., in everyday face-to-face communication); at other times, they can be achieved by deliberate analysis of the viewed face (e.g., when training to recognize deception, as described above). Whereas dual-systems accounts propose that two qualitatively and architecturally distinct cognitive processing systems underlie judgments in one form or another, a unified approach, such as the adaptive toolbox, proposes that intuitive and deliberate judgments rely on the same (or similar) rules, while differing only along continua of their operating characteristics such as automaticity, awareness/consciousness, or intention. Our results diverge from the broadly held view that intuitive and deliberate judgments are formed via two qualitatively and architecturally distinct cognitive processing systems. General dual-systems models assume that various dichotomies (i.e., associative versus rule-based; automatic versus non-automatic) map onto each other but importantly, also that the two thus created systems can operate without the each other (Keren and Schul, 2009). The results of the conjunction- and covariate analyses as well as the direct group comparison all speak against this notion. The following arguments shall clarify this conclusion in more detail.

### Predictions of a Dualistic Paradigm: Two Distinct Systems

Results of all three analyses (conjunction, covariate, and direct contrast) speak against predictions derived from the most widely accepted dual-systems view, according to which we should find distinct neuro-cognitive processing systems (in terms of an X- and C-system). However, contrary to these dual-systems predictions, we did not find distinct activation patterns – in terms of an X- and a C-system – for either the direct contrast of processes, nor for the covariate analyses. All in all, we conclude that our data do not fit the predictions of a dualistic distinction, at least in a face judgment paradigm.

### Predictions of a Unified Model: A Common Network with Differential Additions

As stated previously, if the predictions derived from a unified model more closely represents a mapping of people’s judgment and decision-making processes, neural activation patterns could be expected to show the following characteristics: first, an at least partially common network (the regions of which are task dependent) and second, a potential differentiation between the processes, either purely by expansion (more or more highly activated areas for one strategy as opposed to the other), or by differentiation along specific operating continua, such as consciousness. We find a common network of activation in the conjunction analysis, comprising the fusiform gyrus, the cuneus, and the inferior occipital gyrus. Thus both groups engaged the core areas of face perception (Fusar-Poli et al., 2009; Atkinson and Adolphs, 2011) and therefore presumably engaged the same (cognitive) processes when judging the authenticity of emotional facial expressions. We assume, relying on previously well-established results on face processing, that participants in both groups processed second-order relational or configural cues (the metric distance between features) in the faces, reflected by activation in inferior occipital gyrus and fusiform gyrus (Rhodes et al., 2009).

A limitation of our study certainly lies in the higher inter-group variability within the deliberately instructed participants. Although individual differences are tough to control (and actually constitute valuable components of study), future studies that are not expecting OFC activations may be better able to deal with this caveat by raising the global power of the study by measuring gradient-echo sequences. Seeing as we expected activation of the OFC for the intuitive group, and because of its proximity to tissue interfaces this area is known to be susceptible to signal loss caused by magnetic susceptibility differences, we needed to employ spin-echo EPI. Although less sensitive to BOLD functional signal, SE sequences have been proposed as potentially beneficial option to obtain increased functional localization to the capillary bed (Chiacchiaretta and Ferretti, 2015).

However, even though the areas of overlap revealed by the separate covariate analyses did not survive the conjunction threshold (presumably due to high variability of the deliberate group), the tendency for the activation of similar neural substrates in both of the groups is shown. Furthermore, while our data cannot wholly support the unimodel predictions as the “correct” theory, the activations elicited by the direct probing of intuition and deliberation in our every-day type task do not map onto the separable and distinct two systems of judgment in the brain proposed by dual-systems theorists.

The results of our covariate analyses diverge from dual-systems predictions, by showing largely overlapping networks for both the fast and the slow responding individuals in both instruction groups. In detail, instead of finding distinct networks of activation, as would be predicted by a dual-systems account, our covariate analyses show that for both groups similar neural substrates are negatively correlated with RT for the processing of our socially relevant face stimuli, namely regions of primary visual perception [cuneus (BA 17), lingual gyrus] as well as the right OFC, fusiform gyrus, and STG/TPJ. That is, the faster participants’ responded the more these regions were engaged. In contrast to this, the X-system (i.e., reflexive/intuitive) is proposed to involve the amygdala, basal ganglia, and lateral temporal cortex, while the C-system (reflective/deliberate) is thought to rely on the activation of anterior cingulate cortex, the prefrontal cortex, and the medial temporal lobe (Lieberman, 2007).

Based on this divergence and the activation of partially similar networks regardless the induction of differential judgment strategy modes (intuition/deliberation) in our task, we take these results to indicate that very similar cognitive processes may underlie the two decision processes. When judging facial expressions, both seem to be relying on classic areas of face perception (cuneus, lingual gyrus, and fusiform gyrus) as well as on areas of the extended face perception network that are also known to be important for social cognition (OFC and TPJ). Together with the inferior frontal gyrus and medial and lateral parts of the OFC, the TPJ has been shown to be involved in perspective taking during emotional narrative comprehension (Mano et al., 2009). Furthermore, both the OFC as well as the TPJ are involved in visual discrimination as well as person perception and emotional mimicry tasks. It might be that – in order to intuitively or deliberately judge the authenticity of emotional facial expressions – people try to take the perspective of the person they are seeing. In a sense, they might be trying to “feel what the other person is feeling”—much like the involvement of somatosensory areas in the viewing of emotional faces (Rizzolatti and Craighero, 2004). How much of a role exactly emotional mimicry plays in this task is a question to be answered in future studies. Thus, these results support the notion that both intuition and deliberation share a common (neural) denominator, rather than being architecturally distinct brain systems as proposed by dual-systems proponents (e.g., Sloman, 2014).

While intuition and deliberation share some of the same neural structures when judging the authenticity of emotional faces as presented in this work, some divergent additions to the common network were shown. Particularly, clusters of activation around the TPJ observed in the intuitive group extended into the posterior part of the STS, which has also been shown to represent perceived emotions at an abstract, modality-independent level (Peelen et al., 2010), along with being one of the areas involved in face perception and communication (Adolphs, 2003; Kanwisher and Yovel, 2006). Facial mimicry plays a prominent role in the judgment of another’s facial expression and the simulation of the perceived emotion in oneself. In what way facial mimicry might play a role for intuitive processing (but perhaps not in deliberate one), is a question beyond the purview of the present study. It would be interesting to investigate whether a mimicry response might differ between an intuitive and a deliberate judgment condition. The literature thus far gives mixed indications for the use of mimicry in the judgment of genuineness of smiles. Hess et al. (1998) found that just evoking the notion that expressions may not be genuine, by asking participants to rate their genuineness, eliminated mimicry to these expressions. When participants rated the emotion rather than the genuineness of the same expressions, they were mimicked. However, Rychlowska et al. (2014) showed that blocking mimicry compromised the decoding of true and false smiles. It is therefore unclear if the question of authenticity, employed in the current work, would evoke mimicry in either an intuitive or a deliberate condition^2^. Since the onset of a smile has been shown to be important in the judgment of this expression (e.g., Carr et al., 2014), future studies will surely benefit from the use of dynamic facial expressions and concurrent facial EMG measurements.

The neural network of the intuitive group additionally engaged the left OFC, IPL, precuneus and anterior insula bilaterally, which were not evident in the deliberate group. Activation within the anterior (agranular) insula – which is most closely connected to the (medial) OFC – has been suggested as a “part of a core of a system for evaluating primary reinforcers and determining appropriate motivational states – that is, core affect” (Wager and Barrett, 2004, p. 7). This re-representation of interoception is seen as a possible basis for the anterior insula’s involvement in subjective (self-relevant) feelings (Critchley et al., 2004; Craig, 2009) and thus may support the “awareness of the immediate moment with a coherent representation of ‘my feelings’ about ‘that thing”’ (Craig, 2009, p. 65). Thus, the anterior insula via its connection to the OFC may instantiate the (gut) feeling that we suppose to arise from the non-conscious cognitive processing being indicative for the intuitive decision mode. A crucial role for these non-conscious cognitive processes is assigned to the OFC. This area has strong reciprocal functional connections to virtually all sensory modalities: it is a gateway between the limbic system (by way of a coupling process involving the hypothalamus and the amygdala) and representational memory (Zald and Rauch, 2006). This anatomical characteristic may underlie the subconscious involvement of pre-existing knowledge in intuitive judgments and decisions of humans—a hallmark of the concept of intuition (Ilg et al., 2007; Volz and Zander, 2014) and may have its effects via the connection to the anterior insula.

Together, the differential activation of the bilateral OFC along with the anterior insula in the intuitive condition may reflect the dimension of non-conscious processing as a distinguishing factor for intuitive judgment. However, consciousness in the realm of judgment processes constitutes an operating characteristic and not a functional characterization of the process itself. This is of importance, since dual-systems theories often confound operating principles (i.e., the mental mechanisms that translate inputs into outputs) with operating characteristics (i.e., the characteristics of or conditions in which a mental process operates, e.g., speed, awareness, intention, or control; see Ferguson et al., 2014; Gawronski et al., 2014 for an overview of these criticisms). Stating that a process operates in an automatic or non-automatic fashion simply specifies when the process is assumed to operate; it does not specify how the process translates inputs into outputs. Continuous operating characteristics (such as consciousness) cannot be combined to form two discrete systems (Ferguson et al., 2014). In fact, two of the most prominent researchers on conscious and unconscious processing recently joined forces and proclaimed that: “Whatever we may have thought and seemed to say in the past, at present we both think that most human behavior comes from a blend of conscious and unconscious processes working together to meet the person’s critical needs and facilitate important goal pursuits” (Baumeister and Bargh, 2014). Conceptually, intuition is often associated with an (evolutionarily) “old” system in the brain that is thought to be engaged automatically and non-consciously. However, when we speak of non-conscious processing here, we are not referring to reflexive behavior, which occurs without conscious awareness of the acting agent. We use the term non-conscious to refer to the observation that people are not, or only partly aware of the cognitive processes underlying their judgment, in the sense that they cannot report on them. For instance, they cannot report on the cues they are using or on the way in which these cues are processed (cp. Volz and Zander, 2014). We refer to the conceptualization of intuition which assumes that people – when engaged in intuitive processing – act based on a strong “feeling” (see Proust, 2015 on the structure and function of these feelings) without being able to consciously reason about the origin of this feeling or the cognitive basis thereof (Glöckner and Witteman, 2010; Kruglanski and Gigerenzer, 2011). In other words, the non-conscious processing (of which the decision maker is unaware) takes effect through consciously experienced signals [such as “feeling of rightness” (Thompson et al., 2011) or “feeling of (processing) fluency” (related to intuition; see, e.g., Dijkstra et al., 2014)], which are colloquially denoted as gut feelings.

We therefore take the finding of similar neural substrates (right OFC and TPJ, along with core areas of face perception) in both groups for the judgment of emotional facial expressions, as well as the expansion of activation within area’s known to be involved in mentalizing and unconscious processing in the intuitive condition (bilateral OFC and anterior Insula), to contradict popular dual-systems accounts that propose a clear-cut dichotomy of “two distinct systems in the brain that serve complementary functions” (Sloman, 2014). The intuitive decision-maker may not be aware of the reason for deciding in a particular way, but has a strong feeling for deciding that way. We thus believe the anterior insula activation to be at the core of the awareness of the subjective feeling leading the decision-maker to her (intuitive) judgment.

To summarize, we would tentatively suggest that the present work does not match the neural predictions derived from a dual-systems model. Our results seem rather in support a unified model according to which judging intuitively or deliberately both rely on similar or same mental processes following similar processing rules, while differentially engaging divergent mental structures to support (continuous) operating characteristics. As the unimodel suggests, any rule can be easier (or more difficult) to process, used consciously or non-consciously, and its use is not determined by the operation of one distinct system over another but rather the amount of available resources (motivational, cognitive, etc.; Kruglanski and Gigerenzer, 2011). Further support for this view comes from Huang and Bargh’s (2014) proposal that both conscious and unconscious processes may operate on the basis of the same mental structures involving the same mental operations.

Non-verbal signals such as facial expressions have repeatedly been shown to be critical in the shaping of social behavior (e.g., Critchley et al., 2000) and are *“among the most informative stimuli we ever perceive”* (Tsao and Livingstone, 2008). The social complexity hypothesis posits that as social structures grow in complexity, so does the need for more complex forms of cognition and communication. A notion of duality – whether it be the general notion of dual systems or the more specific notion of dual-processes (cp. Chaiken and Trope, 1999; Evans and Stanovich, 2013, for example) has certainly been a great catalyst for the production of a plethora of scientific evidence, especially in decision science, social psychology and (neuro-)economics. Whether these dualisms can truly be applied to explain all of the complexity that is necessitated by life in social structures remains to be seen. For the judgment of faces, at least, the story might be a less dualistic one.

To our knowledge, this work is the first to directly instruct intuitive and deliberative modes in a face judgment task in order to probe the mental mechanisms underlying these processes. It would certainly be interesting to try to extend these findings to a greater population, by employing a larger sample size and utilizing a random effects analysis. Perhaps by increasing the sample size, one might be able to directly investigate sub-groups forming within the deliberate group (as seen from the high inter-group variability in the deliberate condition in the present study) and compare these to an intuitive strategy. Our study can therefore only provide a first stepping stone, for the further development of direct probing into the neural basis of intuitive and deliberative (face) judgments.

## Conflict of Interest Statement

The authors declare that the research was conducted in the absence of any commercial or financial relationships that could be construed as a potential conflict of interest.

## Footnotes

1 Most of cognitive neuroscience studies exclusively study the neural correlates for decisions under risk (e.g., lotteries) and imply that understanding these would lead to an understanding of decision-making in an uncertain world (cp. Volz and Gigerenzer, 2012).

2 Note, however, that these seeming divergences may be the result of task-induced differences. While Hess et al. (1998), asked participants to rate genuineness of smiles, Rychlowska et al. (2014) presented smiles that had been previously marked as “true” or “false” and measured mimicry to these pre-rated stimuli.
